# Diversity of Microbial Communities and Quantitative Chemodiversity in Layers of Marine Sediment Cores from a Causeway (Kaichu-Doro) in Okinawa Island, Japan

**DOI:** 10.3389/fmicb.2017.02451

**Published:** 2017-12-11

**Authors:** Taha Soliman, James D. Reimer, Sung-Yin Yang, Alejandro Villar-Briones, Michael C. Roy, Holger Jenke-Kodama

**Affiliations:** ^1^Microbiology and Biochemistry of Secondary Metabolites Unit, Okinawa Institute of Science and Technology Graduate University (OIST), Kunigami, Japan; ^2^Molecular Invertebrate Systematics and Ecology Laboratory, Graduate School of Engineering and Science, University of the Ryukyus, Nishihara, Japan; ^3^Genetics and Genetic Engineering Research Group, National Institute of Oceanography and Fisheries (NIOF), Cairo, Egypt; ^4^Tropical Biosphere Research Center, University of the Ryukyus, Nishihara, Japan; ^5^Biodiversity Research Center, Academia Sinica, Taipei, Taiwan; ^6^Imaging and Instrumental Analysis Section, Okinawa Institute of Science and Technology Graduate University (OIST), Kunigami, Japan

**Keywords:** bacteria, archaea, eukaryotes, metagenomics, NGS, chemodiversity

## Abstract

Microbial community diversity and chemodiversity were investigated in marine sediments adjacent to the Okinawan “Kaichu-Doro” Causeway, which was constructed 46 years ago to connect a group of four islands (Henza-jima, Miyagi-jima, Ikei-jima, Hamahiga-jima) to the Okinawan main island. This causeway was not built on pilings, but by land reclamation; hence, it now acts as a long, thin peninsula. The construction of this causeway was previously shown to have influenced the surrounding marine ecosystem, causing ecosystem fragmentation and loss of water circulation. In this study, we collected sediment cores (*n* = 10) from five paired sites in 1 m water depths. Each pair of sites consisted of one site each on the immediate north and south sides of the causeway. Originally the members of each pair were much closer to each other (<150 m) than to other pairs, but now the members of each pair are isolated by the causeway. Each core was 60–80 cm long and was divided into 15-cm layers. We examined the vertical diversity of microbial communities and chemical compounds to determine the correlation between chemodiversity and microbial communities among marine sediment cores and layers. Principal coordinate analyses (PCoA) of detected compounds and of bacterial and archaeal operational taxonomic units (OTUs) revealed that the north and south sides of the causeway are relatively isolated, with each side having unique microbial OTUs. Additionally, some bacterial families (e.g., Acidaminobacteraceae, Rhizobiaceae, and Xanthomonadaceae) were found only on the south side of Kaichu-Doro. Interestingly, we found that the relative abundance of OTUs for some microbial families increased from top to bottom, but this was reversed in some other families. We conclude that the causeway has altered microbial community composition and metabolite profiles in marine sediments.

## Introduction

Anthropogenic impacts such as pollution, large-scale coastal construction, and overexploitation of marine resources have affected many marine ecosystems (Islam and Tanaka, 2004), including those of the shallow waters in the Ryukyu Archipelago in southern Japan (Reimer et al., 2015). Okinawa-jima, the main island, located in the central Ryukyu Archipelago, is situated in a marine area with high biodiversity that is under threat from anthropogenic impacts (Roberts et al., 2002). The causeway “Kaichu-Doro” is one example of large-scale coastal development on Okinawa. This causeway was built about 46 years ago (1971–1974) to connect four smaller islands (Henza-jima I., Miyagi-jima I., Ikei-jima I., Hamahiga-jima I.) to Okinawa, effectively cutting a tidal mud flat into two separate areas, a northern side in Kin Bay and a southern side in the Pacific Ocean (Reimer et al., 2015, Figure 1).

**Figure 1:**
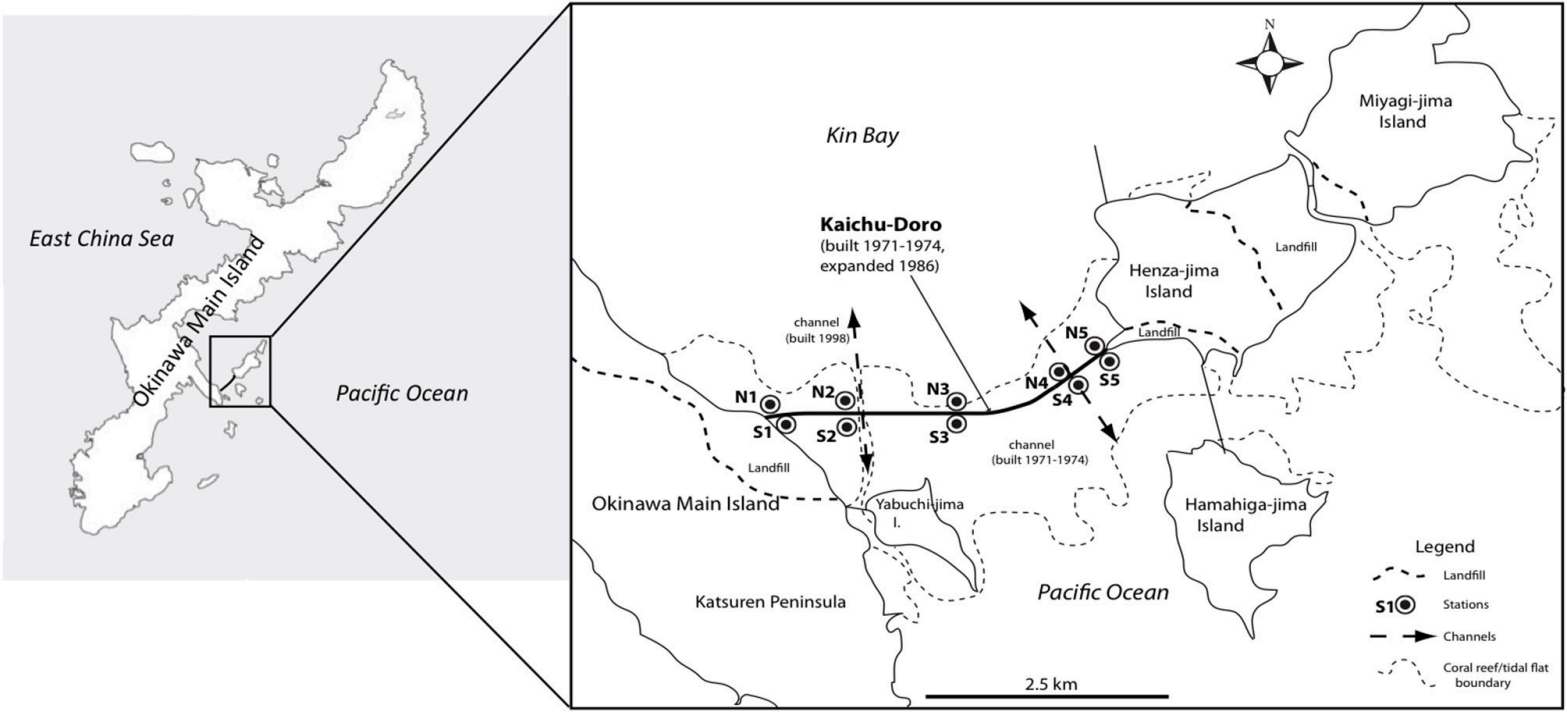
Sampling sites at the causeway, Kaichu-Doro, Okinawa Main Island, Japan (adapted from Reimer et al., 2015).

Microbial communities and their metabolites are critical to the health of marine ecosystems (Das et al., 2006). Previous studies have reported the importance of marine microbes in mangrove ecosystems in photosynthesis (Vethanayagam, 1991), nitrogen fixation (Toledo et al., 1995), methanogenesis (Mohanraju and Natarajan, 1992), and the production of antibiotics and enzymes (Kathiresan and Bingham, 2001). In other ecosystems, such as coral reefs, microbial communities in marine sediment are a source of phosphorus and nitrogen, and corals support bacterial activity by producing mucus, which contains proteins and polysaccharides (Wild et al., 2004).

In general, marine sediment contains numerous microbes (bacteria, archaea, fungi, and other eukaryotes) and it has been challenging to estimate their abundance and distribution in sediments. However, recently, next generation sequencing (NGS) has allowed acquisition of DNA sequences from various sediments with increasing accuracy (Quail et al., 2012), and studies on microbial diversity within marine sediments have accelerated (e.g., Acosta-Gonzalez and Marques, 2016; Fan and Xing, 2016; Galand et al., 2016; Hu et al., 2016; Stagars et al., 2016; Walsh et al., 2016).

The term chemodiversity is generally used to describe the diversity of chemical compounds in a specified context. Chemodiversity studies are often restricted to a certain group of organisms, such as *Aspergillus* species (Frisvad and Larsen, 2015) or to a specific set of plant secondary metabolites (Moore et al., 2014). However, chemodiversity really embraces all metabolites present in complex environments, like soil or water. For instance, recent studies have analyzed the influence of soils on wines produced from two proximate vineyards in the Bourgogne area of France (Roullier-Gall et al., 2014) and climate-dependent differences in the distribution of dissolved organic matter in lakes (Kellerman et al., 2014). Here, we define chemodiversity as the aggregate of all detectable chemical compounds within marine sediment cores, without focusing on particular producers or subsets of natural products.

The aim of this study was to follow-up on a previous investigation by Reimer et al. (2015), which showed differences in taxonomic diversity and abundance on either side of the Kaichu-Doro causeway. In this study, we specifically investigated compositional differences of microbial communities (bacteria, archaea, fungi and other eukaryotes) and quantitative chemodiversity across a vertical gradient within 1 m sediment cores taken from various sites along the causeway. We then examined the correlation between chemical compounds and microbial communities among marine sediment layers. The results of the present study will be useful not only to furnish baseline marine microbe diversity data for the Kaichu-Doro causeway, but can also serve as a model for examining long-term impacts of coastal construction on marine sediment microbial communities.

## Materials and methods

### Cores

Marine sediment cores were collected as described in Reimer et al. (2015) from the north (N1–N5) and south (S1–S5) sides or “coasts” of Kaichu-Doro (Figure 1). Cores were approximately 60–80 cm in depth (Figure 1), and were transported and stored at −80°C at the University of the Ryukyus, Okinawa, Japan. Core sediments were separated into vertical layers ~15 cm in length (total *n* = 47) and sediment dating was based on sedimentation rates (Reimer et al., 2015), and designated N1A (north core 1 layer A), N1B (north core 1 layer B), etc. (A–F from top to bottom). Additional information about cores, such as sampling dates, time, characteristics of sediments, and a full description of the study area were detailed in Reimer et al. (2015).

### DNA extraction and PCR

Sediments of each layer were mixed well. Triplicate samples of total genomic DNA were extracted from 0.5 g of each layer of core sediment using a MO BIO PowerSoil® DNA Isolation kit (MO BIO Laboratories, Carlsbad, CA, USA) following the manufacturer's protocol. Polymerase chain reaction (PCR) amplifications were conducted using 16S rRNA gene primers [341F, 5′-CCTACGGGNGGCWGCAG-3′; 805R, 5′-GACTACHVGGGTATCTAATCC-3′ (Herlemann et al., 2011)] for the V3-V4 region of bacteria, 16S rRNA gene primers [340F, 5′-CCCTAYGGGGYGCASCAG-3′ (Gantner et al., 2011); 915R, 5′-GTGCTCCCCCGCCAATTCCT-3′ (Stahl and Amann, 1991)] for archaea, internal transcribed spacer (ITS) region primers [ITS3, 5′-GCATCGATGAAGAACGCAGC-3′; ITS4, 5′-TCCTCCGCTTATTGATATGC-3′ (White et al., 1990)] for fungi, and 18S rRNA primers [1380F, 5′-CCCTGCCHTTTGTACACAC-3′; 1510R, 5′-CCTTCYGCAGGTTCACCTAC-3′ (Amaral-Zettler et al., 2009)] for other eukaryotes. PCR amplifications were conducted in 20 μL total volume containing 4 μL (10 ng/μL) of microbial template genomic DNA, 0.6 μL (10 mM) of each forward and reverse primer, 4.8 μL of PCR-grade water, and 10 μL of 2 × KAPA HiFi HotStart ReadyMix (Kapa Biosystems, Boston, MA, USA). PCR conditions were 95°C for 5 min (initial denaturing), 30 cycles of 20 s at 98°C, 20 s at optimum annealing temperatures, and 30 s at 72°C, and a final extension at 72°C for 5 min. Amplicons were confirmed and sized using gel electrophoresis (1.2% and 1 × TAE buffer). PCR products were cleaned up using AMPure XP beads (Agencourt ®AMPure ®XP kit, Beckman Coulter, USA), according to the Illumina MiSeq protocol for amplicon preparation. Amplicon products were sequenced using the Illumina MiSeq platform at the DNA Sequencing Section of the Okinawa Institute of Science and Technology (OIST) Graduate University (Onna, Okinawa, Japan).

### Methanol extraction of samples

One gram of sediment sample (wet weight) was transferred to a 15-mL Falcon tube and twice extracted with 5 mL methanol. The extraction was carried out by vortexing (30 s), and sonication (10 min), followed by centrifugation (10 min, 9,000 g, 10°C). Pooled methanol extracts were transferred to a new Falcon tube (15 mL) and dried in a vacuum concentrator (40°C, TOMY Speed Vac). One mL of methanol was added to the resulting dried extract, vortexed (1 min), and sonicated (10 min). Finally, the suspension was transferred to an Eppendorf tube (2 mL) and centrifuged (10 min, 14,000 g, 10°C) to give a clear methanol solution. The clear methanol solution was decanted in an Eppendorf tube (2 mL) and dried in the vacuum concentrator (40°C). The resulting extract was finally dissolved in methanol (200 mL) and centrifuged (10 min, 14,000 g, 10°C) to give a clean methanol extract. The methanol extract was either analyzed immediately or stored at −30°C.

### Liquid chromatography-mass spectrometry (LC-MS)

A Thermo Scientific Mass Spectrometer (Q Exactive Plus, Bremen, Germany) was used for mass spectrometry (MS) data collection. The mass spectrometer was equipped with an ultra-high-pressure liquid chromatography (UHPLC) (Dionex Ultimate 3000, Thermo Scientific), an auto-sampler (HTC PAL, CTC Analytics, Zwingen, Switzerland), and a heated electrospray ionization source-II (H-ESI). Two tandem spectra were generated for each targeted compound in positive ion mode. H-ESI probe conditions were set as follows: sheath gas flows 35, auxiliary gas flow 10, sweep gas flow 0, spray voltage 3.5 kV, capillary temperature at 290°C, S-lens RF level at 70.0, and auxiliary gas heater at 350°C. Full MS spectra were acquired at 70,000 resolution AGC target 3e^6^, maximum IT 100 ms, and full mass range m/z 250–1,200 Da. Data-dependent MS^2^ spectrum was configured to select top ions from the MS^1^ spectrum, with 35,000 resolution, AGC target 1e^5^, maximum IT 50 ms, loop count 5, optimized collision energy (NCE 35), and isolation window (4.0 m/z).

Clean samples were separated on an Ascentis Express C_18_ column (150 × 2.1 mm, 2.7 μm, Supelco). An 18-min step-gradient was used for compound separation (10% B for 0.0-2.0 min, 10 to 25% B for 2.0–2.5 min, 25 to 100% B for 2.5–10.0 min, hold 100% B until 14.0 min, equilibration 10% B from 14.1 to 18.0 min, where solvent A was aqueous-acetonitrile 95:5 and solvent B was aqueous-acetonitrile 10:90. Both solvents contained 0.1% formic acid. A flow rate of 300 μL/min was used and the column temperature was maintained at 40°C). A 15 μL (20 μL loop) sample was injected using the auto-sampler and triplicate runs were carried out for each sample.

### Data analyses

The Illumina MiSeq (V3 kit) produced 2 × 300 bp paired-end sequences, and quality control of raw fastq data files was performed using FastQC v0.11.4 (Andrews, 2010). High-throughput sequence raw data were imported into CLC Genomics Workbench version 9.0.1 with plugins CLC Microbial Genomics Module version 1.3.1 (QIAGEN, Aarhus A/S, http://www.clcbio.com) according to quality scores from Illumina pipeline 1.8, in order to achieve the highest quality sequences for clustering. Sequence data were trimmed using 0.05 as a limit for quality scores with 2 as the maximum number of ambiguities. The optional merge paired reads method was run with default settings (mismatch cost = 1; minimum score = 40; gap cost = 4 and maximum unaligned end mismatch = 5). Sequence reads were clustered and chimeric sequences detected using an identity of 97% as the Operational Taxonomic Unit (OTU) threshold. Reference OTU data used in the present study were downloaded from the Greengenes database (DeSantis et al., 2006) for 16S rRNA (bacteria and archaea), the Unite database (Koljalg et al., 2013) for ITS (fungi), and Silva (Quast et al., 2013) for 18S rRNA (eukaryotes). Relative abundances of the most abundant OTUs among all four-sediment layers for all 10 cores were estimated at the family level for archaea, bacteria, eukaryotes, and fungi. OTU relative abundances are presented in separate figures for high and low abundance values of each microbial community based on the number of sequences per OTU. High-throughput sequencing data from this study were deposited in the Sequence Read Archive (SRA) of GenBank under Accession number SRP077849. LC-MS raw data were processed and analyzed using Mzmine version 2.02 (Pluskal et al., 2010), XCMS R package (Smith et al., 2006) and MetaboAnalyst version 3.0 (Xia et al., 2015). Our strategy for analyzing the quantitative chemodiversity of sediment cores was to perform an initial general screening of methanol extracts by mass spectrometry (LC-MS) without focusing on specific metabolites or compound groups and without attempting to identify the compounds. The initial screening resulted in thousands of peaks, as would be expected for an environment as complex as marine sediment. In order to reduce the complexity of analyses, we selected the most significant MS signals based on *p*-values (*p* ≤ 0.01) and Relative Standard Deviations (RSD ≤ 10%) among layers of sampled cores. This procedure resulted in a selection of 195 peaks (see Table S1).

Overall, Good's estimator of coverage [(1–(singletons/individuals)) × 100] and Shannon's entropy were estimated for all microbial communities in the present study. A Permutational Multivariate Analysis of Variance (PERMANOVA) test was conducted based on Bray-Curtis (Beals, 1984) and Weighted UniFrac (Lozupone et al., 2011) distances among cores, and separately among the top layer (A). PERMANOVA parameters were measured using 100,000 permutations using CLC Genomics Workbench version 9.0.1 and the data grouped based on layers (AN, BN, CN, DN, EN, AS, BS, CS, DS, ES, FS), cores (N1–N5 and S1–S5) and locations (north and south). Principal Coordinates Analysis (PCoA) was used to characterize all layers and the top layer (A) among cores based on Bray-Curtis for both community structure and chemodiversity. Additionally, alpha diversity (rarefaction curves) was calculated based on Chao 1 measurements. PCoA and Alpha diversity were conducted for microbial communities using CLC Microbial Genomics Module version 1.3.1. Partial Least Squares Discriminant Analysis (PLS) estimated the weighted sum of absolute regression coefficients among the microbial communities from the top layers (A) of the cores. The heatmap visualization was estimated based on Euclidean matrix distances, and Ward's clustering algorithm was utilized for chemodiversity features and OTU abundances as identified by *t*-test analysis (*p* < 0.05) among all layers. Seawater environmental data obtained by Reimer et al. (2015) were interpreted and discussed with microbial community top layers (A).

## Results

### Numbers of gene sequences and coverage

Numbers of predicted operational taxonomic units (OTUs) defined by nucleotide sequence identity higher than 95% were estimated for all communities (bacteria = 6,039; archaea = 517; fungi = 580; other eukaryotes = 764). Predicted OTUs, reads in OTUs, filtered reads, Good's coverage, and Shannon entropy values of each layer are summarized in Table 1. All layers showed a Good's sequencing coverage of at least 95%. Characteristic changes in OTU-based diversity, as measured by the number of different OTUs and Shannon entropy could not be observed for most of the cores. Only in cores N5 and S2 did the diversity of all communities steadily increase from the top to the bottom layers. In general, the diversity of fungal and other eukaryotes were much more constant among layers compared to bacteria and archaea.

**Table 1:** Summary of reads, operational taxonomic unit (OTU) numbers, and overall parameters of microbial communities from sites at Kaichu-Doro, Okinawa, Japan.

**ID**	**Bacteria**					**Archaea**					**Fungi**					**Other eukaryotes**				
	**OTUs**	**Reads in OTUs**	**Filtered reads**	**Good's coverage**	**Shannon entropy**	**OTUs**	**Reads in OTUs**	**Filtered reads**	**Good's coverage**	**Shannon entropy**	**OTUs**	**Reads in OTUs**	**Filtered reads**	**Good's coverage**	**Shannon entropy**	**OTUs**	**Reads in OTUs**	**Filtered reads**	**Good's coverage**	**Shannon entropy**
N1A	594	12,702	67,371	95.33	8.00	218	32,759	79,739	99.33	5.33	33	15,589	87,248	99.79	4.23	118	34,988	121,974	99.66	4.4
N1B	399	13,995	82,964	97.15	7.41	122	43,815	79,218	99.72	3.69	27	12,784	92,333	99.79	3.96	81	12,088	106,339	99.33	3.59
N1C	390	16,484	86,481	97.63	7.37	111	44,896	87,563	99.75	3.78	22	16,609	87,319	99.87	3.8	95	20,304	65,449	99.53	3.57
N1D	374	14,705	74,872	97.46	6.95	94	40,633	96,548	99.77	3.75	28	9,962	101,632	99.72	3.84	149	37,384	82,297	99.60	3.82
N2A	622	14,694	65,126	95.77	8.19	217	25,372	83,451	99.15	5.25	37	12,271	66,239	99.70	4.32	95	23,000	104,156	99.59	4.63
N2B	601	15,843	66,698	96.21	7.97	217	23,192	88,569	99.06	5.32	59	17,292	79,254	99.66	4.5	116	12,589	80,747	99.08	4.7
N2C	544	16,912	70,676	96.78	8.07	208	28,215	87,726	99.26	4.69	52	15,698	96,573	99.67	4.31	128	25,362	71,985	99.49	4.79
N2D	484	19,890	70,382	97.56	7.69	175	33,303	89,260	99.47	3.97	29	6,363	107,552	99.54	3.92	112	34,397	113,134	99.67	3.99
N2E	418	20,560	68,625	97.97	7.21	153	23,664	69,150	99.35	4.22	34	1,659	66,876	97.98	4.1	119	29,409	142,134	99.60	4.02
N3A	477	14,444	56,385	96.70	6.81	171	21,210	58,431	99.20	4.67	76	10,938	60,292	99.31	5.02	118	30,897	38,913	99.62	3.96
N3B	493	11,269	57,261	95.63	7.41	190	19,048	60,974	99.00	5.03	28	12,129	74,927	99.77	4.08	96	30,425	71,497	99.69	4.43
N3C	564	12,159	61,764	95.36	7.87	201	16,692	63,433	98.79	5.43	54	18,027	71,301	99.70	4.74	107	34,777	69,217	99.69	3.14
N3D	588	16,327	64,679	96.40	7.89	194	16,641	64,752	98.83	5.3	29	3,032	63,355	99.04	4.19	98	38,255	59,072	99.74	2.74
N4A	585	20,802	69,068	97.19	7.58	193	20,139	65,124	99.04	4.98	52	8,177	75,986	99.36	4.93	84	32,190	42,945	99.74	1.07
N4B	577	16,498	58,778	96.51	7.61	182	12,272	52,152	98.52	5.14	64	9,878	57,343	99.35	4.97	107	16,786	50,862	99.36	3.1
N4C	554	14,109	50,882	96.07	7.60	145	9,216	35,712	98.42	4.5	51	2,066	37,325	97.54	4.77	92	29,964	49,313	99.69	3.39
N4D	434	17,054	64,270	97.45	6.76	179	30,529	80,530	99.42	4.47	23	9,514	83,036	99.76	3.73	101	33,535	74,285	99.70	4.85
N4E	444	16,824	64,854	97.36	6.83	170	29,415	79,338	99.42	4.27	52	11,495	75,327	99.55	4.7	102	40,226	93,547	99.75	4.53
N5A	381	30,363	52,834	98.75	6.29	135	40,753	80,935	99.67	4.33	45	13,745	84,007	99.67	4.46	71	19,851	48,147	99.64	4.47
N5B	389	29,066	56,269	98.66	6.62	150	34,134	77,508	99.56	4.43	46	11,839	70,507	99.61	4.63	86	27,507	72,682	99.69	4.88
N5C	584	27,282	57,976	97.86	5.53	176	30,763	77,345	99.43	4.69	28	17,237	64,922	99.84	4.25	101	21,246	52,693	99.53	4.35
N5D	564	41,807	58,402	98.65	4.12	196	27,574	82,244	99.29	4.61	48	13,609	48,390	99.65	4.8	109	50,535	69,497	99.78	2.16
S1A	461	21,457	59,558	97.85	7.32	176	27,054	73,351	99.35	4.9	37	7,010	76,698	99.47	4.27	106	24,941	65,696	99.58	4.74
S1B	391	27,930	47,405	98.60	7.09	138	35,052	72,321	99.61	4.67	30	7,039	86,134	99.58	4.34	84	26,463	65,487	99.68	4.77
S1C	513	16,595	58,276	96.91	7.41	216	26,390	75,944	99.18	4.91	71	10,807	66,203	99.34	5.38	114	23,432	78,842	99.51	5.34
S1D	520	16,054	62,365	96.76	7.76	229	22,537	74,258	98.98	5.13	74	14,817	44,349	99.50	5.35	121	23,296	81,227	99.48	5.33
S1E	502	22,162	74,604	97.74	7.63	260	28,195	83,778	99.08	5.91	89	16,430	46,722	99.46	5.17	167	24,084	82,473	99.30	5.68
S1F	396	20,941	73,124	98.11	6.75	203	27,285	82,882	99.26	5.12	127	16,855	35,552	99.24	5.72	143	32,059	164,463	99.56	5.22
S2A	242	30,003	47,816	99.19	6.62	91	33,952	60,076	99.73	4.85	29	3,296	47,288	99.13	4.16	103	11,754	34,603	99.13	4.77
S2B	611	16,256	63,233	96.24	7.84	194	25,280	72,633	99.23	4.94	56	10,183	34,645	99.45	5.01	135	21,647	86,318	99.37	4.98
S2C	613	15,046	60,856	95.93	7.72	194	22,843	70,830	99.15	4.99	52	14,682	48,509	99.64	4.57	106	10,733	72,921	99.01	5.28
S2D	632	15,886	58,301	96.02	7.68	172	18,414	65,559	99.06	4.99	74	11,448	50,618	99.36	4.91	148	15,178	78,065	99.02	5.32
S2E	530	27,768	65,935	98.09	6.80	171	11,020	67,222	98.45	5.03	89	9,197	51,555	99.03	4.32	142	31,867	131,269	99.55	2.76
S3A	506	15,061	61,116	96.64	7.32	211	21,951	68,036	99.04	5.25	67	13,183	51,043	99.49	5.11	92	17,727	62,078	99.48	4.97
S3B	447	12,808	64,720	96.51	7.12	182	22,017	69,326	99.18	4.97	71	7,705	48,003	99.08	5.38	90	12,265	59,641	99.27	4.92
S3C	473	8,400	39,699	94.37	7.26	205	19,105	66,037	98.93	5.31	60	9,973	49,776	99.40	4.89	40	4,375	79,761	99.09	3
S3D	351	24,102	57,873	98.55	7.41	101	31,368	53,899	99.68	5.2	52	7,400	49,234	99.30	4.86	68	20,847	58,341	99.67	4.47
S3E	472	22,763	63,473	97.93	7.91	103	35,398	48,228	99.71	4.78	66	8,572	51,993	99.22	4.95	91	45,320	125,323	99.80	4.54
S4A	409	25,192	65,888	98.37	5.44	183	25,192	61,153	99.27	4.8	32	1,546	43,587	97.94	4.44	147	18,384	84,631	99.20	4.83
S4B	331	12,132	68,486	97.27	6.70	106	29,612	58,411	99.64	4.35	31	4,660	91,660	99.34	4.29	140	23,315	146,638	99.40	5
S4C	503	23,651	91,263	97.87	6.12	179	44,233	75,215	99.60	4.88	28	6,199	60,658	99.55	4.19	141	26,332	86,723	99.47	4.69
S4D	606	21,848	86,179	97.23	6.89	223	33,121	77,058	99.33	5.4	18	11,038	125,972	99.84	3.62	102	9,841	60,776	98.97	5.04
S4E	598	16,488	73,268	96.37	7.59	220	34,361	80,802	99.36	5.2	49	12,462	70,010	99.60	4.76	88	36,965	181,135	99.76	4.66
S5A	601	17,357	79,361	96.54	7.72	232	22,900	78,202	98.99	5.7	63	11,684	61,327	99.46	5.01	102	23,972	109,375	99.57	4.65
S5B	592	14,071	66,715	95.79	7.89	211	25,885	76,422	99.19	5.47	57	10,748	50,843	99.47	4.87	134	35,315	95,194	99.62	4.58
S5C	620	16,286	71,848	96.19	7.68	218	31,353	76,648	99.30	5.24	60	10,454	57,412	99.43	4.72	159	39,413	154,632	99.60	5.24
S5D	639	21,170	75,622	96.98	7.34	200	27,132	66,682	99.26	5.19	51	11,359	61,357	99.55	4.76	96	83,466	83,256	99.89	2.65

### Bacterial and archaeal communities

**Figure 2:**
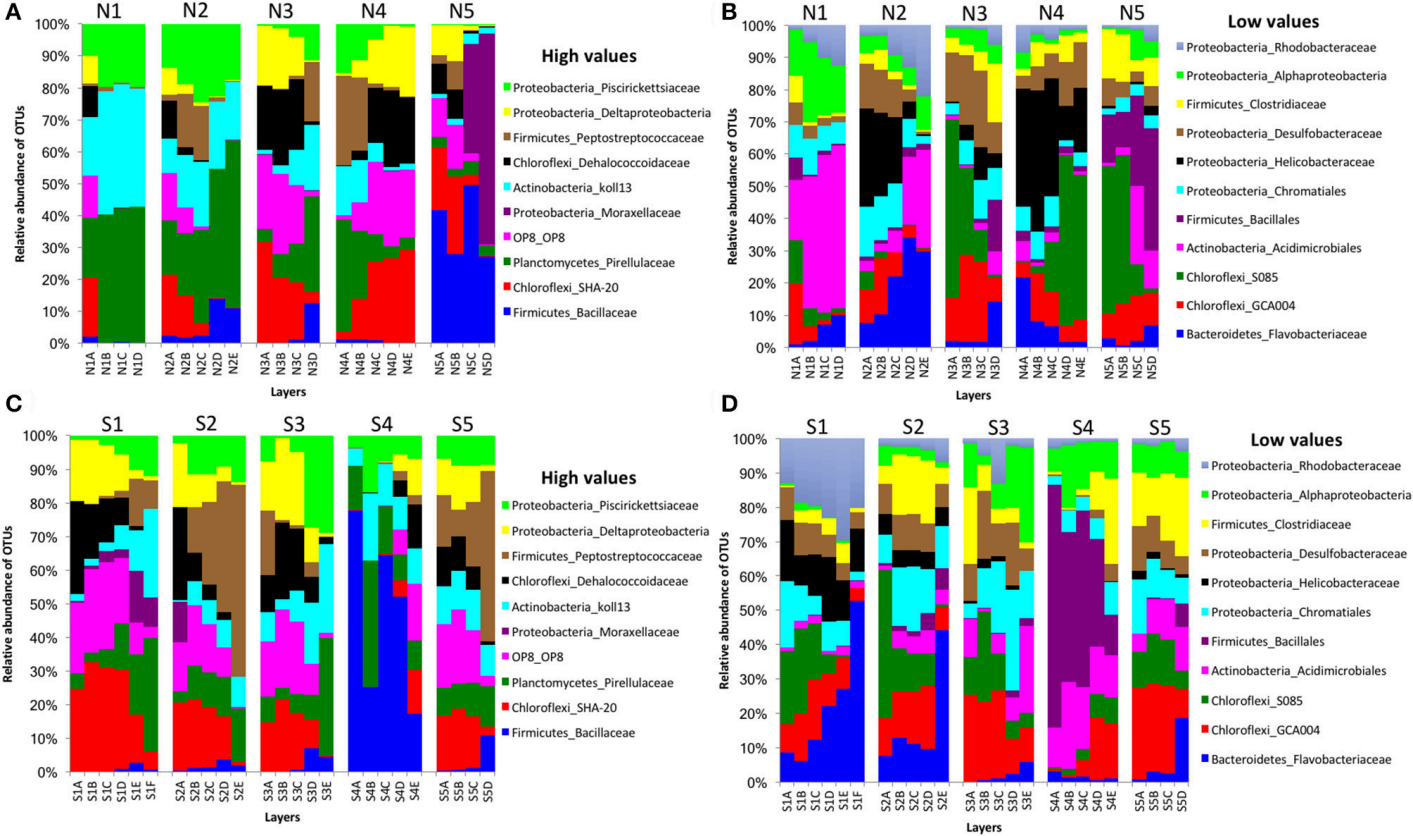
Relative abundances of bacterial OTUs between north (N) and south (S) sites at Kaichu-Doro among layers (A–F). High **(A)** and low **(B)** OTU abundance on the north; high **(C)** and low **(D)** OTU abundances values on the south.

In bacterial communities, the relative abundance of OTUs of the class Deltaproteobacteria (phylum Proteobacteria) clearly decreased from top to bottom layers in cores N2, N3, S1, S2, and S3, but this trend was reversed in core N4 (Figures 2A,C). Likewise, the Atribacteria (formerly called candidate phylum OP8) and SHA-20 showed the same patterns of distribution among layers (Figures 2A,C). However, other phyla such as Firmicutes, Actinobacteria, Chloroflexi, Cyanobacteria, Planctomycetes, and Bacteroidetes showed fluctuations in OTU values among all layers from both the north and south sides of the causeway (Figures 2C,D). In cores S4 and N4, the photosynthetic Chloroflexi showed an unusual distribution as their proportions increased in deeper layers. The family Bacillaceae was abundant in cores N5 (49%) and S4 (38%) and the order Bacillales (unclassified family) was high in the same two cores (N5 and S4) compared to other cores (Figures 2C,D). In addition, the family Helicobacteraceae (Proteobacteria) made up the highest percentages of OTUs in cores N2, N4, and S1 (Figures 2B,D). In addition, the family Moraxellaceae (Proteobacteria) showed high relative abundance in layers N5C-D of core N5 (Figure 2A). Unique OTUs of bacterial phyla Bacteroidetes (order: Bacteroidales), Proteobacteria (order: Rhizobiales; families: Rhizobiaceae, Rhodobacteraceae, Pseudomonadaceae, Xanthomonadaceae; genera: *Rhodobacter, Paracoccus, Thiothrix, Dechloromonas*), and Verrucomicrobia (genus: *Luteolibacter*) were present only in cores from the south side of Kaichu-Doro (Table 2).

**Table 2:** Unique operational taxonomic units (OTUs) of 47 marine sediment layers of microbial communities among layers between the north and south sides of Kaichu-Doro, Okinawa, Japan.

**Domain**	**OUT_Name**	**Taxonomy**	**North**	**South**	**AN**	**AS**	**BN**	**BS**	**CN**	**CS**	**DN**	**DS**	**EN**	**ES**	**FS**
Bacteria	850572	Proteobacteria_f_Rhizobiaceae	0	236	0	12	0	35	0	32	0	46	0	70	41
	750263	Proteobacteria_o_Rhizobiales	0	265	0	8	0	81	0	26	0	49	0	54	47
	656520	Proteobacteria_g_*Rhodobacter*	0	312	0	15	0	37	0	46	0	59	0	102	53
	593437	Proteobacteria_f_Rhodobacteraceae	0	323	0	10	0	23	0	71	0	58	0	90	71
	582083	Proteobacteria_g_*Paracoccus*	0	1,135	0	67	0	233	0	134	0	189	0	262	250
	540561	Verrucomicrobia_g_*Luteolibacter*	0	316	0	0	0	2	0	41	0	81	0	93	99
	509023	Bacteroidetes_o_Bacteroidales	0	472	0	18	0	20	0	78	0	131	0	224	1
	349981	Proteobacteria_f_Pseudomonadaceae	0	208	0	22	0	21	0	72	0	63	0	30	0
	266510	Proteobacteria_f_Xanthomonadaceae	0	257	0	9	0	18	0	35	0	53	0	88	54
	102284	Proteobacteria_g_*Thiothrix*	0	669	0	82	0	83	0	84	0	114	0	170	136
	646970	Proteobacteria_g_*Dechloromonas*	0	249	0	3	0	36	0	40	0	61	0	101	8
	667356	Firmicutes_g_*Fusibacter*	0	469	0	19	0	11	0	71	0	118	0	215	35
Archaea	561472	Euryarchaeota_g_*Methanosaeta*	0	159	0	4	0	0	0	4	0	10	0	74	67
	194137	Euryarchaeota_g_*Methanobacterium*	0	118	0	0	0	1	0	0	0	3	0	20	94
Fungi	SH000838.07FU_FJ430713	Ascomycota_*Acremonium* sp.	0	509	0	0	0	0	0	0	0	97	0	380	32
	SH025172.07FU_AB220280	Ascomycota_*Apiosporaceae* sp.	0	290	0	0	0	0	0	209	0	81	0	0	0
	SH028501.07FU_GU721693	Ascomycota_*Ascochyta hordei*	0	241	0	0	0	0	0	124	0	0	0	0	117
	SH021969.07FU_JX045723	Ascomycota_*Ascomycota* sp.	0	576	0	360	0	0	0	215	0	0	0	1	0
	SH013923.07FU_HQ871756	Ascomycota_*Ascomycota* sp.	628	0	628	0	0	0	0	0	0	0	0	0	0
	SH018601.07FU_EF568042	Ascomycota_*Candida tropicalis*	0	346	0	0	0	97	0	0	0	26	0	185	38
	SH027295.07FU_JF499833	Ascomycota_*Catenulostroma hermanusense*	0	311	0	0	0	0	0	0	0	311	0	0	0
	SH028824.07FU_JN698876	Ascomycota_*Cladosporium* sp.	389	0	0	0	0	0	0	0	389	0	0	0	0
	SH008744.07FU_HE861827	Ascomycota_*Curvularia sorghina*	0	690	0	0	0	690	0	0	0	0	0	0	0
	SH012462.07FU_AY163551	Ascomycota_*Exophiala oligosperma*	0	932	0	28	0	0	0	335	0	334	0	106	129
	SH000011.07FU_U17329	Ascomycota_*Magnaporthe grisea*	205	0	42	0	0	0	0	0	163	0	0	0	0
	SH026809.07FU_AB586986	Ascomycota_*Monographella nivalis*	0	277	0	1	0	32	0	244	0	0	0	0	0
	SH023251.07FU_EF626950	Ascomycota_*Penicillium cinnamopurpureum*	0	268	0	173	0	0	0	0	0	88	0	0	7
	SH008750.07FU_JN207275	Ascomycota_*Pleosporaceae* sp.	0	427	0	129	0	0	0	0	0	1	0	297	0
	SH004150.07FU_EF488382	Ascomycota_*Pleosporales* sp.	1334	0	0	0	1	0	829	0	504	0	0	0	0
	SH006395.07FU_HM101040	Ascomycota_*Pyrenochaeta* sp.	0	255	0	0	0	0	0	117	0	86	0	0	52
	SH012463.07FU_EF551461	Ascomycota_*Rhinocladiella similis*	0	252	0	0	0	53	0	0	0	117	0	50	32
	SH008701.07FU_AJ888440	Ascomycota_*Scedosporium aurantiacum*	0	371	0	0	0	1	0	0	0	29	0	0	341
	SH028424.07FU_KC507201	Ascomycota_*Sordariomycetes* sp.	208	0	208	0	0	0	0	0	0	0	0	0	0
	SH031971.07FU_UDB017903	Basidiomycota_*Agaricales* sp.	518	0	1	0	0	0	517	0	0	0	0	0	0
	SH031719.07FU_JN943116	Basidiomycota_*Coprinellus micaceus*	0	266	0	28	0	0	0	238	0	0	0	0	0
	SH021888.07FU_AF145321	Basidiomycota_*Cryptococcus albidus*	0	308	0	0	0	0	0	91	0	0	0	0	217
	SH031997.07FU_JN845192	Basidiomycota_*Geastrum mirabile*	807	0	0	0	201	0	508	0	98	0	0	0	0
	SH032509.07FU_AF145568	Basidiomycota_*Hyphodontia radula*	0	223	0	169	0	0	0	54	0	0	0	0	0
	SH001156.07FU_JQ247380	Basidiomycota_*Lycoperdaceae* sp.	256	0	195	0	0	0	61	0	0	0	0	0	0
	SH009613.07FU_KJ832046	Basidiomycota_*Peniophora* sp.	234	0	0	0	0	0	0	0	234	0	0	0	0
	SH029696.07FU_DQ647498	Basidiomycota_*Peniophorella odontiiformis*	264	0	19	0	0	0	0	0	245	0	0	0	0
	SH003649.07FU_JN048775	Basidiomycota_*Polyporaceae* sp.	210	0	0	0	0	0	210	0	0	0	0	0	0
	SH009609.07FU_DQ350127	Basidiomycota_*Russulales* sp.	648	0	0	0	0	0	647	0	0	0	1	0	0
	SH010416.07FU_UDB011478	Basidiomycota_*Schizophyllum commune*	0	396	0	0	0	323	0	73	0	0	0	0	0
	SH013736.07FU_AY015439	Basidiomycota_*Sporobolomyces ruberrimus*	0	224	0	0	0	0	0	113	0	0	0	111	0
	SH027576.07FU_UDB011756	Basidiomycota_*Tapinella atrotomentosa*	241	0	0	0	0	0	241	0	0	0	0	0	0
	SH014060.07FU_AF444439	Basidiomycota_*Trichosporon ovoides*	0	1264	0	31	0	0	0	74	0	888	0	202	69
Other Eukaryotes	AF436003.1.1814	Opisthokonta_*Neotrypaea californiensis*	0	247	0	45	0	196	0	4	0	2	0	0	0
	KC529425.1.1823	Opisthokonta_*Kymocarens* sp. NVS-2013	0	324	0	0	0	264	0	30	0	30	0	0	0
	GU295204.1.1763	SAR_*Thecadinium kofoidii*	684	0	0	0	0	0	37	0	647	0	0	0	0

**Figure 3:**
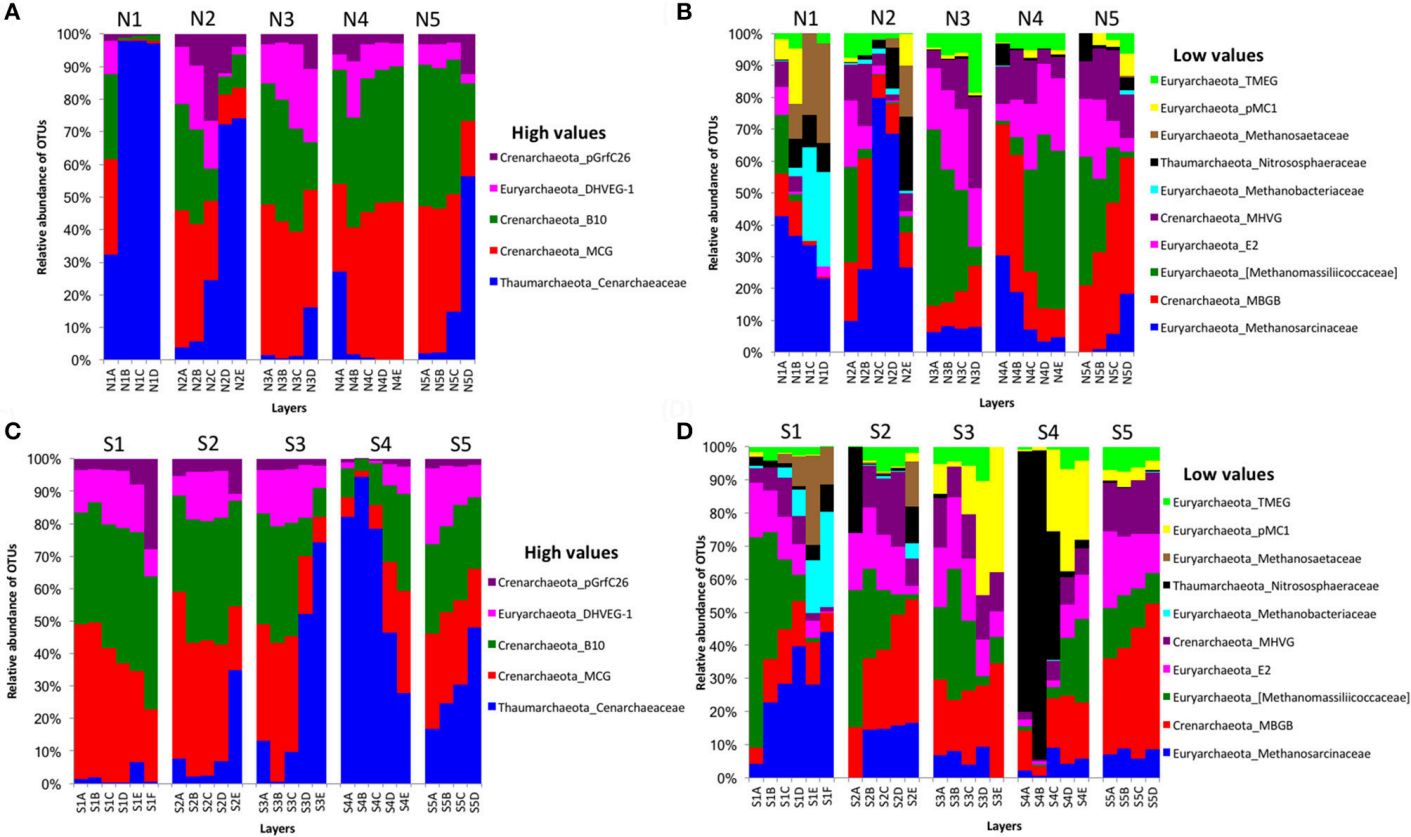
Relative abundances of archaeal OTUs between north (N) and south (S) side sites at Kaichu-Doro among layers (A–F). High **(A)** and low **(B)** OTU abundance on the north; high **(C)** and low **(D)** OTU abundances values on the south.

For archaeal communities, we obtained 1,285,885 archaea-specific reads out of a total of 3,394,675 filtered sequence reads. The diversity of archaeal OTUs was lower than the diversities found for other organism groups. The dominant phyla were Crenarchaeota, Euryarchaeota, and Thaumarchaeota (Table 1 and Figure 3). OTUs in the family Crenarchaeaceae were most abundant in cores N1, N2 and S3-S5, and the family Nitrososphaeraceae was abundant only in core S4 in the top layers (Figure 3D). Group pMC1 of the phylum Euryarchaeota had the highest relative abundance in layers C-E of cores S3 and S4 (Figure 3D). OTUs of the phylum Euryarchaeota varied from top to bottom for cores from both sides (from 9 to 22%; Figures 3A–D). Unique OTUs of the genera *Methanosaeta* and *Methanobacterium* were only seen on the south side of Kaichu-Doro (159 and 118 sequences, respectively; Table 2).

### Fungal and other eukaryotic communities

**Figure 4:**
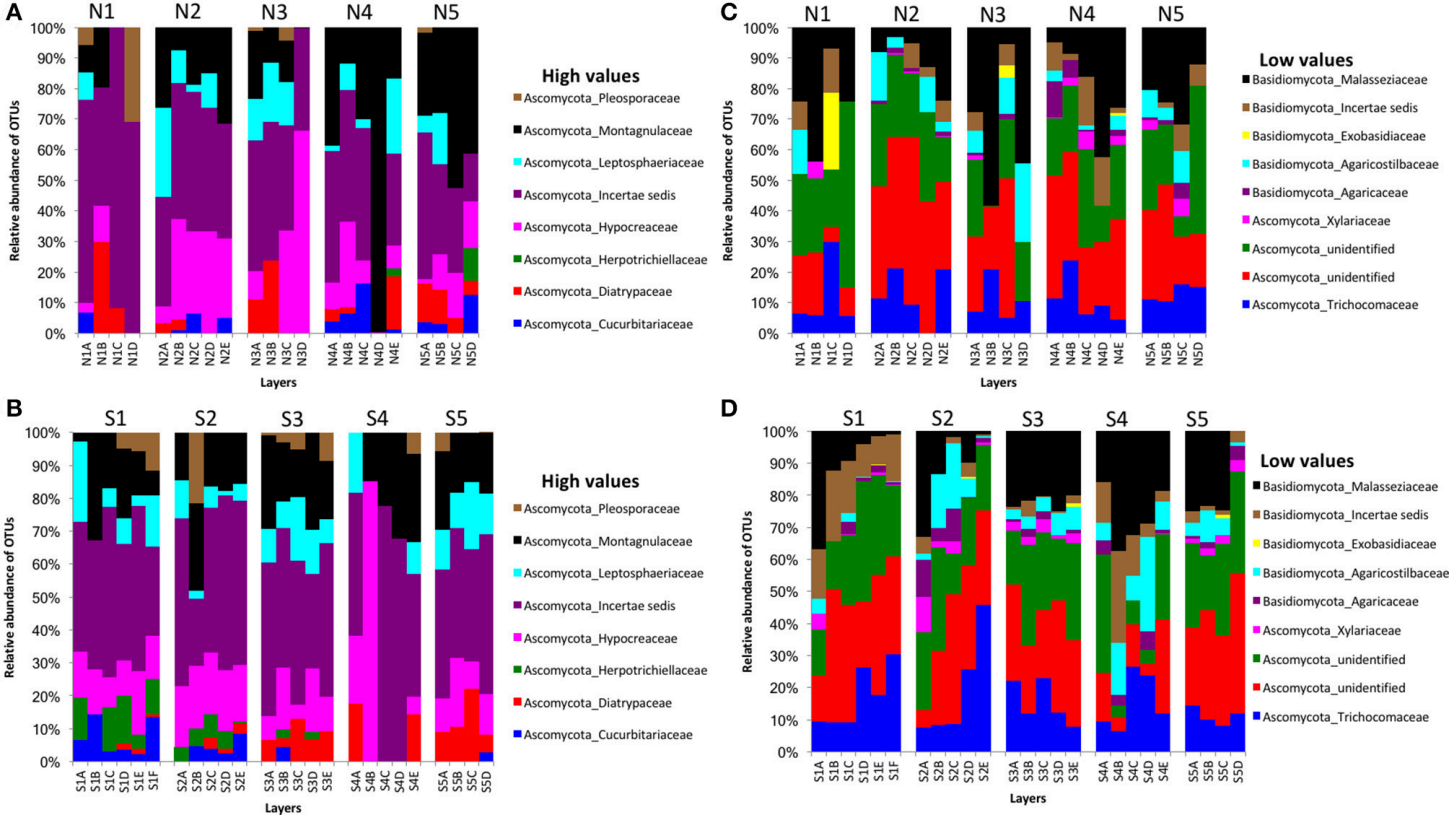
Relative abundances of fungal OTUs between north (N) and south (S) side sites at Kaichu-Doro among layers (A–F). High **(A)** and low **(B)** OTU abundance on the north; high **(C)** and low **(D)** OTU abundances values on the south.

We obtained 498,660 fungal reads and 580 total predicted OTUs from a total of 3,112,892 filtered sequence reads targeting the ITS-3 and ITS-4 regions (Table 1). Fungal OTU abundances indicated variations among layers from cores on both sides of Kaichu-Doro (Figures 4A–D). Incertae sedis were abundant among all layers of all cores (Figures 4A,C). A relatively high abundance of OTUs of the family Exobasidiaceae was present in N1C and N3C with small abundances in other cores (Figures 4B,D). We estimated 13 and 20 unique OTUs among fungal taxa from the north and south sides of Kaichu-Doro, respectively (Table 2).

**Figure 5:**
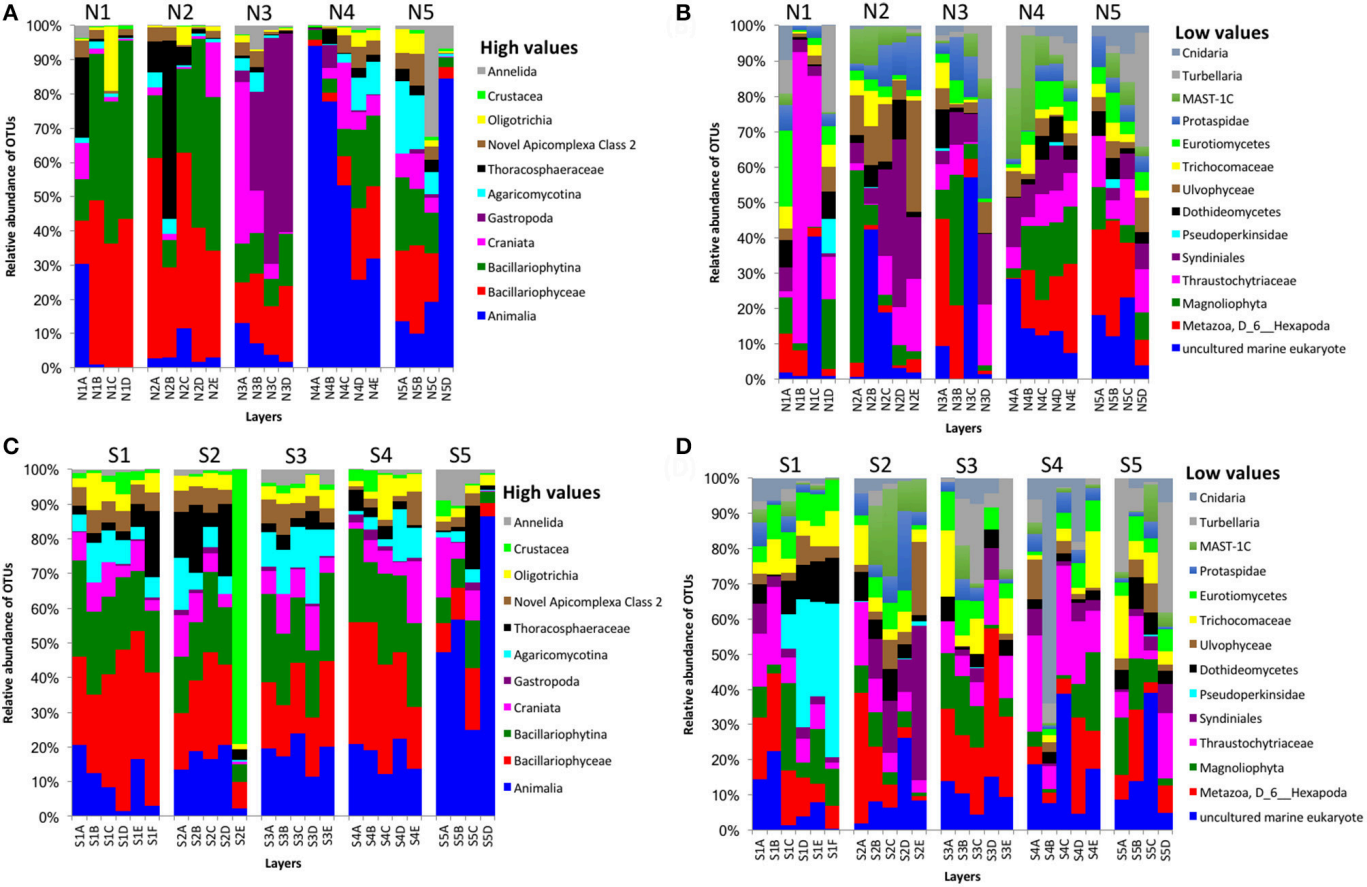
Relative abundances of other eukaryotes OTUs between north (N) and south (S) side sites at Kaichu-Doro among layers (A–F). High **(A)** and low **(B)** OTU abundance on the north; high **(C)** and low **(D)** OTU abundances values on the south.

Partial sequencing of the eukaryotic 18S rRNA gene produced 764 total predicted OTUs, comprising 1,278,706 specific reads from a total of 4,009,753 filtered reads (Table 1). The relative abundance of eukaryote OTUs, such as Animalia, Bacillariophyceae, Bacillariophytina, Gastropoda, and Crustacea increased from top to bottom on the north side of Kaichu-Doro, and this pattern was reversed for Agaricomycotina, Craniata, Novel Apicomplexa Class 2, and Thoracosphaeraceae. Gastropoda and Crustacea were abundant in cores N3B-D and S2 (among all layers), (Figures 5A,C). In addition, the relative OTU abundance of Pseudoperkinsidae was high in cores S1, layers C-F from the south side, and in Core N1 layer D (Figures 4B,D). *Neotrypaea californiensis* (ghost shrimp, 247 sequences) and *Kymocarens* sp. NVS-2013 (flatworm, 324 sequences) were unique to the south side of Kaichu-Doro, and *Thecadinium kofoidii* (dinoflagellate, 684 sequences) was unique to the north side (Table 2).

### Statistical analyses of community structure and chemodiversity

To test statistically whether there were differences in structures of microbial communities with regard to location, we performed permutational multivariate analyses of variance (PERMANOVA) for each type of community among layers and cores, and compared community composition of north and south sites as well. Differences in overall layers of all communities were insignificant (*p* values; bacteria = 0.8612, archaea = 0.5020, fungi = 0.3577 and other eukaryotes = 0.2945), but were significant in chemodiversity analyses (*p* < 0.002). This means that there was no clear vertical trend for changes in community composition. In contrast, the PERMANOVA test of composition of community structure and chemodiversity among all cores showed significant differences (*p* < 0.0001). However, the total comparison between north and south showed significant differences for other eukaryotic communities and chemodiversity (*p* < 0.002), but not for bacteria (0.0887), for archaea (0.3423), or for fungi (0.1315). Additionally, PERMANOVA was used to test only layer A among all north and south sites. It revealed insignificant differences (*p*-values; bacteria = 0.8174, archaea = 0.8095, fungi = 0.2698, other eukaryotes = 0.8968).

### Sea water environment and microbial communities in the top layer

**Figure 6:**
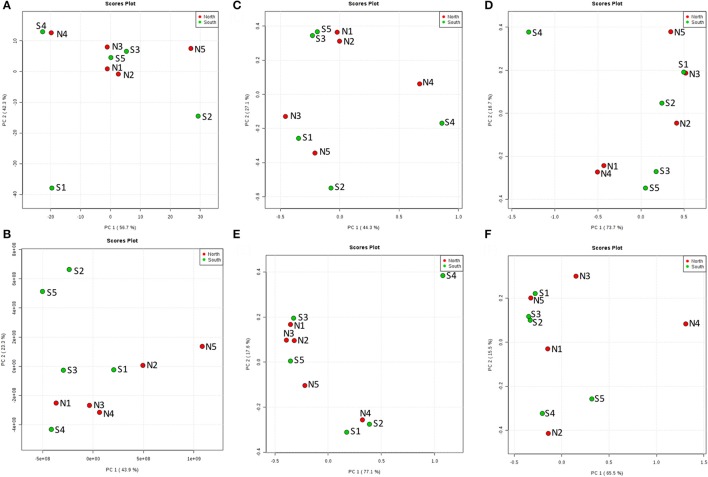
Principal Coordinates Analysis (PCoA) of the top layer (A) among 10 cores for north and south sides of the Kaichu-Doro causeway. **(A)** Environmental factors; **(B)** chemodiversity (LC-MS); **(C)** bacteria; **(D)** archaea; **(E)** fungi; **(F)** other eukaryotes.

From the PCoA result of seawater environmental data (Reimer et al., 2015), sites N1, N2, N3, S3, and S5 had similar features. S4 and N4 were similar, and S1, S2, and N5 were distinct from other groups (Figure 6A). However, chemodiversity (Figure 6B) showed different patterns, with north and south sites separated, except for S4, which resembled north sites.

**Figure 7:**
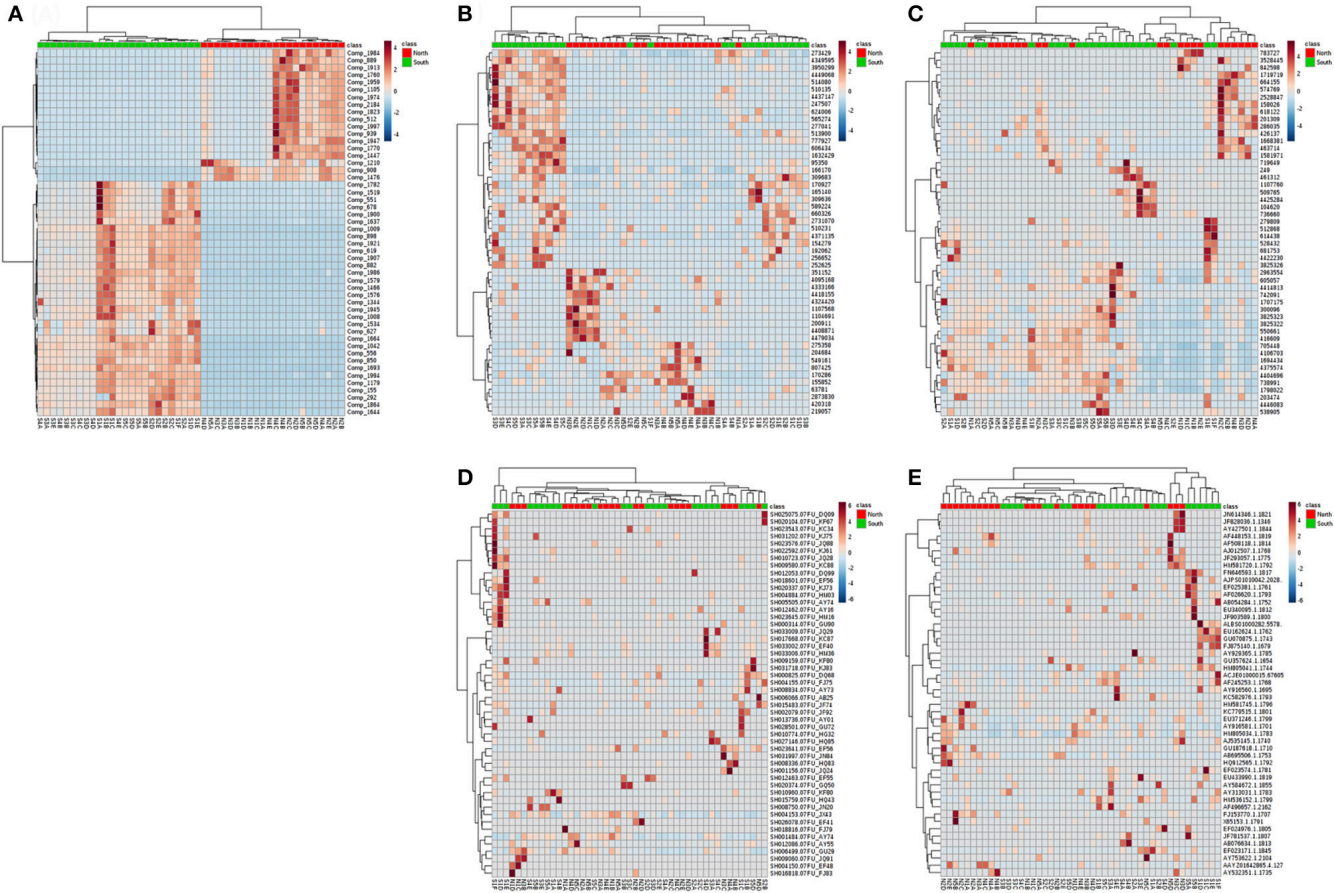
Heatmap for the hierarchical clustering (using Euclidean matrix distances, and Ward's clustering algorithm) for chemodiversity features and OTU abundances among all layers of the Kaichu-Doro causeway. **(A)** Metabolites; **(B)** bacteria; **(C)** archaea; **(D)** fungi; **(E)** other eukaryotes.

Similar patterns could be found throughout all layers. Although the number of compounds and microbial communities per layer did not show clear differences within or among cores, Principle Coordinate Analysis (PCoA) of compounds indicated two relatively discrete clusters corresponding to the north and south sides of the causeway among all layers (Figure S1A). Similarly, PCoA showed the same general clustering pattern for the OTUs of overall bacterial and archaeal communities (Figures S1B,C). In contrast, PCoA revealed no pattern of clearly clustered groups among fungal and other eukaryote OTUs (Figures S1D,E). While targeting high abundance OTUs and metabolites, differences of south and north were more pronounced (Figures 7A–E). Metabolite classes from north and south were very distinct (Figure 7A) throughout layers and sites. The distribution of high-abundance bacterial OTUs was also bimodal (Figure 7). In addition, it spread into five clusters: (1) S3, S4 and S5; (2) N1C, D, N2D, E, and N3D; (3) N2 to N5, with S1F and S2E from the deepest layers of south cores; (4) N1A, B, and S4A, B; (5) S1, S2 and S3B. For other dominant OTUs in archaea (Figure 7C), fungi (Figure 7D) and eukaryotes (Figure 7E), there were no clear groupings for sites or layers. Alpha rarefaction curves were constructed for each core sample showing numbers of OTUs (Chao 1), based on minimum numbers of reads (Figure S2). Core N5 showed low numbers of OTUs of archaea and other eukaryotes, but core S4 showed the highest number of OTUs in both communities (Figures S2B,D). However, core S5 presented the lowest number of OTUs of bacteria, archaea, and fungi (Figures S2A–C).

**Figure 8:**
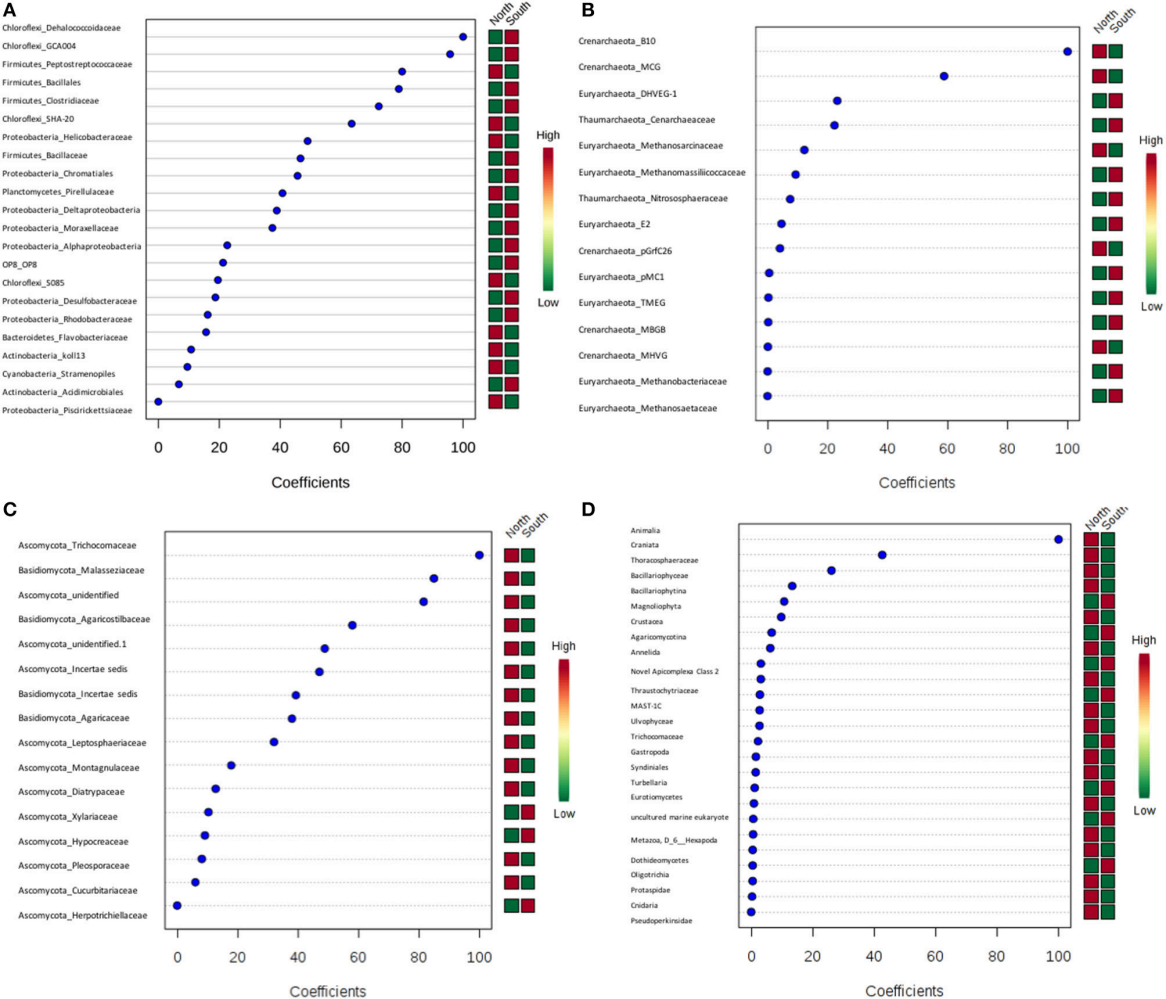
Partial least squares Discriminant Analysis (PLS-DA) based on weighted sum of absolute regression coefficients. Colored boxes on the right indicate the relative abundance of corresponding OTUs in north and south sides. **(A)** Bacteria; **(B)** archaea; **(C)** fungi; **(D)** other eukaryotes.

PLS-DA showed there were certain microbial groups that had strong correlations with the north or south side (Figures 8A–D). For bacteria, two classes from Chloroflexi, Dehalococcoidaceae and GCA004, were mainly found in the south, while of three Firmicutes classes, the Peptostreptococcacea was more common in the north, whereas the Bacillales and Clostridiaceae were found more in the south. For archaea, there were only two Crenarchaeota, predominately found in the north. Trichocomaceae and one unidentified class of fungi from Ascomycota and class Basidiomycota from Malasseziaceae were identified more frequently in the north (Figures 8A–D).

## Discussion

Microbial communities have previously been investigated from shallow to deep ocean waters, as well as from marine sediments using next-generation sequencing (NGS) (DeLong et al., 2006; Brown et al., 2009; Hu et al., 2016; Walsh et al., 2016). Recently, high-throughput sequencing technology was used to examine microbial communities in deep-sea sediments and to investigate sediment-hosted CO_2_ in the Okinawa Trough (Inagaki et al., 2006; Zhang J. et al., 2015). However, despite such applications of this technology, many ecosystems throughout the world remain to be investigated. In the present study, we examined the vertical distribution of microbial communities from marine sediment cores. Another novelty of the current study was the analyses of chemodiversity along the causeway to investigate possible correlations between microbial communities and compounds detectable in sediment cores.

Our study indicates that some groups of organisms may have relatively definite depth profiles based on the vertical composition of microbial communities in the cores, with either an increase in abundance from top to bottom, or vice-versa. Such patterns were particularly common in bacterial communities (Figures 2A–D). Previously, most studies have reported on variations of microbial community composition in the water column and gyres, with various results by depth (Brown et al., 2009; Treusch et al., 2009; Ghiglione et al., 2012; Fan and Xing, 2016; Walsh et al., 2016). However, the present study focused on the diversity of microbial communities among marine sediment layers on a small geographic scale in shallow water. Therefore, we expected that our results would diverge from those of previous studies due to habitat differences and specific characteristics of sediment and water. Microbial composition is affected by many environmental factors, including temperature, dissolved oxygen, nutrients, and pH (Baril et al., 2012; Teixiera and Merquior, 2014; Mandic-Mulec et al., 2015). In addition, some other environmental factors have also been identified as important determinants of sediment microbial composition, such as sedimentological characteristics and organic matter content (Dang et al., 2008, 2009, 2013).

Overall, among bacterial communities, the phyla Actinobacteria (21%), Chloroflexi (23%), Firmicutes (25%), and Proteobacteria (18%) were dominant among all samples (*n* = 47) in the present study. Some bacterial families showed a specific pattern within some cores. For instance, the family Bacillaceae and order Bacillales (unclassified family) of the phylum Firmicutes were dominant among all layers of cores N5 and S4 (Figures 2A,C). The reason for the high abundance of Bacillaceae and Moraxellaceae in N5 could be due to a shrimp farm nearby, since these forms commonly dominate shrimp pond water (Vanderzant et al., 1971). The family Bacillaceae is widely distributed in soil, marine sediments, air, food, and other environments (Mandic-Mulec et al., 2015; Krishnamoorthy et al., 2017). It is important in degradation of soil organic matter and in phosphorus solubilization, and it is able to form resistance endospores (Mandic-Mulec et al., 2015). Some studies of soils have reported that members of the family Bacillaceae interact and colonize organic matter (Siala et al., 1974; Toljander et al., 2006; Mandic-Mulec et al., 2015; Krishnamoorthy et al., 2017). The family Moraxellaceae is found in naturally saline environments and is adapted in low temperatures, but little is known about its ecological role, such as its role in the degradation of organic compounds (Wery et al., 2003; Teixiera and Merquior, 2014). The Eubacteria is known for transforming toxic halogenated ethenes (e.g., tetrachloroethene) to non-toxic ethene (Maymo-Gatell et al., 1997). The high abundance of the Chloroflexi family Dehalococcoidaceae found along this causeway indicates that it might be contaminated by industrial organic pollutants, such as halogenated pollutants, as reported by previous studies in another marine environment (e.g., Wang et al., 2016; Zinder, 2016). Sites S1, S2, and N3 have a high abundance of Dehalococcoidaceae in the top layer, which may be a response to wastewater input, or garbage (Reimer et al., 2015). Interestingly, photosynthetic Chloroflexi was distributed in deep layers of cores N4 and S4. Kurladze and Ivanova (2008) reported that photosynthetic Chloroflexi are capable of chemoheterotrophic growth under aerobic conditions and they can switch from anoxic photosynthesis to aerobic respiration. Additionally, we assume that Chloroflexi were detected in the deep layers of the cores and therefore, photosynthetic activity can be excluded as they are much too far away from the photic zone. Chloroflexi sometimes show diel migration in intertidal microbial mats. However, such diel migration is limited to the mm range. One can speculate that the Chloroflexi of cores N4 and S4 permanently live without photosynthetic activity showing a chemoorganotrophic lifestyle, which may be a result of niche differentiation.

The archaeal family Cenarchaeaceae was more abundant in bottom layers than top layers of cores N1-2, N5, and S2-3 and showed a reversed distribution in core S4. In addition, it was the most abundant family in core N1. The families Cenarchaeaceae and Nitrososphaeraceae belong to phylum Thaumarchaeota, and they are found in marine sediments of marginal seas of the western Pacific Ocean (e.g., Dang et al., 2009, 2010, 2013), including the East China Sea (e.g., Dang et al., 2008). The Thaumarchaeota are chemolithoautotrophic or mixotrophic ammonia oxidizers, playing very important roles in nitrification and inorganic carbon fixation among marine Cenarchaeaceae (Dang and Chen, 2017). The Methanosarcinaceae was distributed among all ten cores and in all four layers of sediment, and was the most abundant group in three cores (N1, N2, and S1). Additionally, the Methanobacteriaceae and Methanosaetaceae were most abundant in cores N1 and S1. Previous studies have demonstrated that Methanobacteriaceae and Methanosarcinaceae are predominant in wastewater treatment sludge, and can adapt to high carbon concentrations (Fei et al., 2015; Kuroda et al., 2015; Fan and Xing, 2016; Hu et al., 2016).

In fungal communities of the present study, Ascomycota were abundant and more frequent than Basidiomycota among all cores. The frequencies of Ascomycota and Basidiomycota shows the similar relative abundance in different climatic origin of marine sediments such as Arctic marine sediments (Zhang T. et al., 2015; Zhang et al., 2016). However, furthermore investigations are needed to address the ecological and biological functions of these fungi, and their adaptations in different environments.

The marine environment may influence some microbial communities in the top layer of the sediment (Figure 6A). However, ocean currents in this area complicate the composition of both macro- and micro-marine biota. The causeway faces a bay to the south with two nearby islands, and it opens to a large bay in north (Figure 1) with tidal currents flowing through channels at sites 2 and 4. The biota surveyed by Reimer et al. (2015) showed variable species diversity and richness along the causeway, which may also influence the composition of microbiota at different sites. Nevertheless, the results of our chemodiversity survey indicate that effects of this causeway have been major, and even with construction of the two channels, differences between the north and south sides are conspicuous (Figure S1, Figure 7) despite their original close proximity. There were more unique OTUs on the south than the north (Table 2). In a previous study, Reimer et al. (2015) reported that while both sides were similar in regard to many marine taxa, there were clear differences in fish communities between the north and south sides, commensurate with our results. Environmental factors may explain some microbial communities at sites N1, N2, S3, and S5 (Figure 6C), archaean communities at sites S3 and S5 (Figure 6D), and fungal communities at sites N1, N2, N3, S3 and S5 (Figure 6E). Eukaryote and other microbial communities (Figure 6F) seem uninfluenced by environmental factors. Despite the channels under the causeway that were built at sites 2 and 4 to mitigate the effect of the causeway, there are few similarities in the environmental factors, chemodiversity, and microbial communities, except at site 4 (Figure 6B).

As a follow-up to Reimer et al. (2015), this study provides crucial baseline information, not only for future changes, but also valuable data for pollution monitoring and control. Based upon environmental data, sites N1, N2, N3, N4, S3, and S4 have been considered less polluted (Reimer et al., 2015), but our data from microbial communities implies there may be pollution. Microbial community composition may have originally been very diverse due to different marine environmental factors; however, chemodiversity results imply that this causeway has caused the south and north sides to differentiate. Finally, *Serratia marcescens*, an opportunistic human pathogen and agent of coral diseases, as previously reported at site S1 (Reimer et al., 2015), and some species of *Bacillus* (Bacillaceae), *Moraxella* (Moraxellaceae), and *Clostridium* (Clostridiaceae), all found in our study, are known to be human and mammalial pathogens. As fishing and recreational activities are popular in the waters around Kaichu-Doro, pollution management is needed to improve water quality and to prevent potential disease outbreaks. Additionally, our results show that even 40+ years after causeway construction the north and south communities show lasting effects of such coastal development.

## Author contributions

TS: design the experimental work, DNA extraction, PCR, LC-MS etc., data analysis, and wrote and review the paper; JR: design the experimental work, sample collection, data analysis, and review the paper; S-YY: data analysis and review the paper; AV-B: experimental work, data analysis (LC-MS), and review the paper; MR: experimental work, data analysis (LC-MS), and review the paper; HJ-K: design the experimental work, sample collection, provide kits, machine and chemicals, data analysis, and review the paper.

### Conflict of interest statement

The authors declare that the research was conducted in the absence of any commercial or financial relationships that could be construed as a potential conflict of interest.

## Supplementary material

Table S1 Raw data for 195 peaks of LC-MS data.

Figure S1 Principal Component Analyses (PCA) of metabolite profiling and overall microbial communities (including 47 samples of various layers). **(A)** compounds; **(B)** bacteria; **(C)** archaea; **(D)** fungi; **(E)** other eukaryotes.

Figure S2 Alpha rarefaction curves representing the number of OTUs (Chao 1) based on the minimum number of reads in each domain of the top layer among 10 cores from the north and the south. **(A)** bacteria; **(B)** archaea; **(C)** fungi; **(D)** other eukaryotes.
