# Supplementary figures and images for: Diversity of Microbial Communities and Quantitative Chemodiversity in Layers of Marine Sediment Cores from a Causeway (Kaichu-Doro) in Okinawa Island, Japan
Source: Front Microbiol. 2017 Dec 11;8:2451. doi: 10.3389/fmicb.2017.02451 (PMC5732179; doi:10.3389/fmicb.2017.02451)

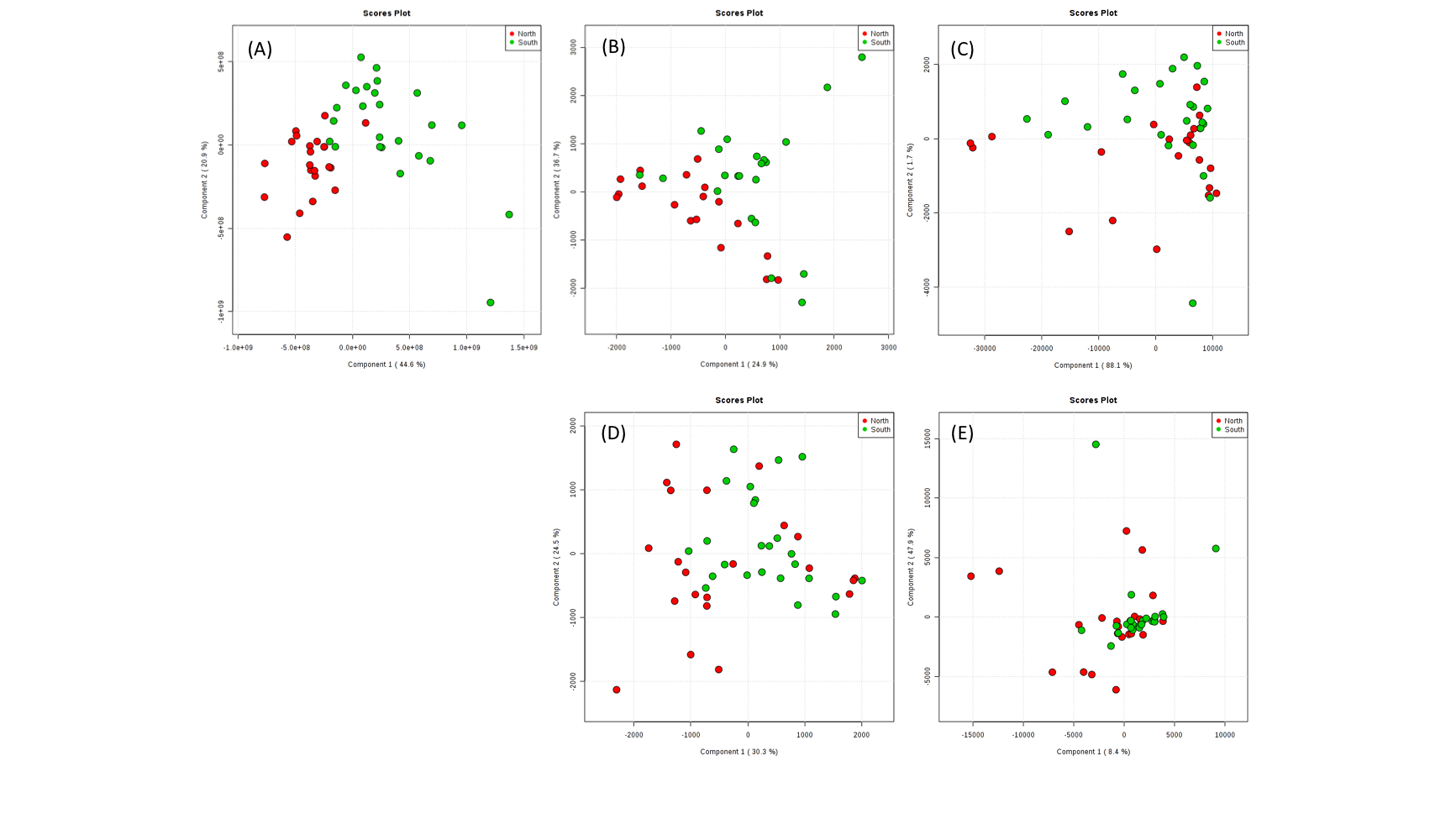

Supplement: Figure S1 — Principal Component Analyses (PCA) of metabolite profiling and overall microbial communities (including 47 samples of various layers). (A) compounds; (B) bacteria; (C) archaea; (D) fungi; (E) other eukaryotes. [file Image1.TIFF]

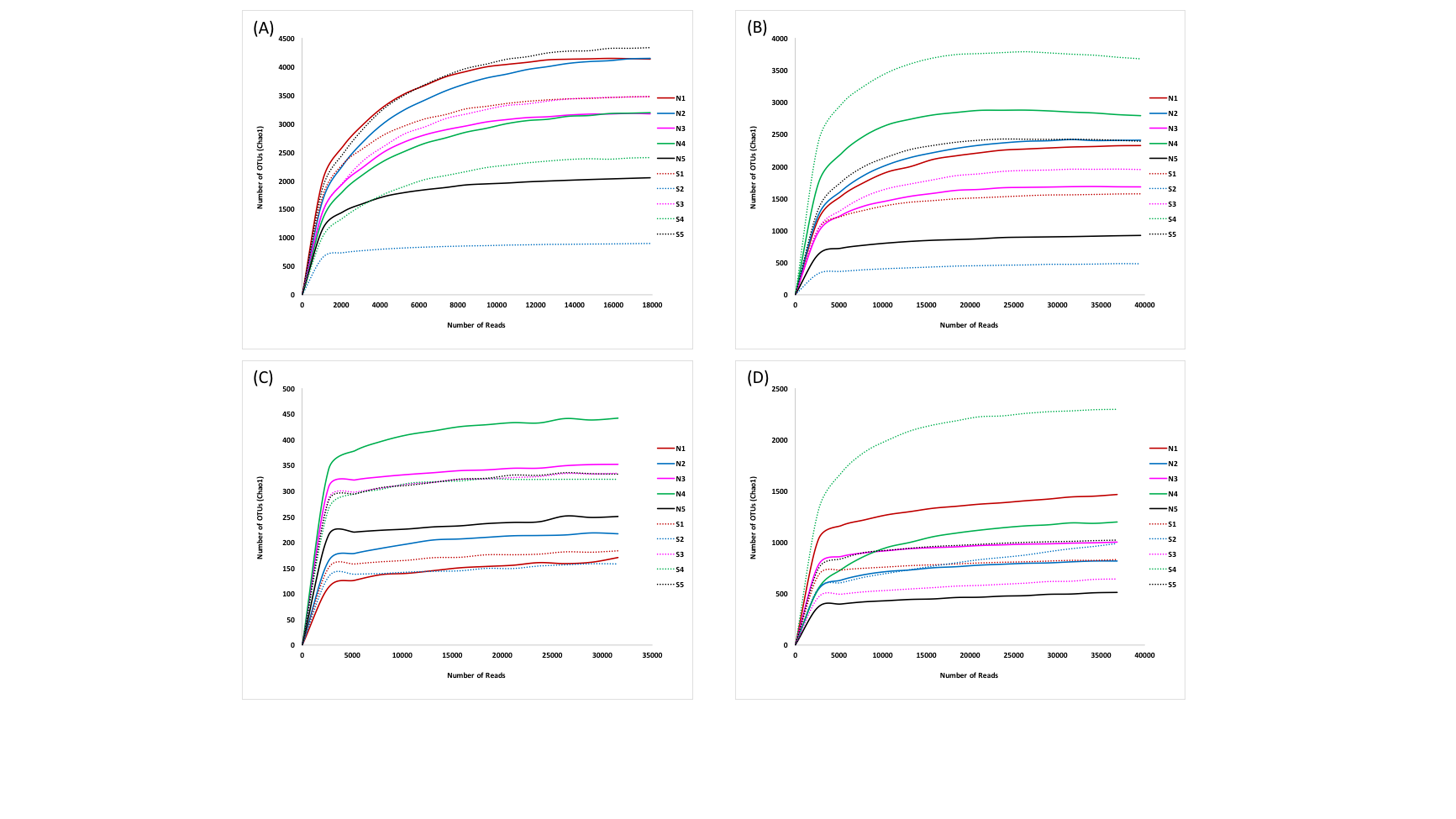

Supplement: Figure S2 — Alpha rarefaction curves representing the number of OTUs (Chao 1) based on the minimum number of reads in each domain of the top layer among 10 cores from the north and the south. (A) bacteria; (B) archaea; (C) fungi; (D) other eukaryotes. [file Image2.TIFF]
